# Temporal Trends in Incidence of Myocardial Infarction and Ischemic Stroke by Socioeconomic Position in Sweden 1987–2010

**DOI:** 10.1371/journal.pone.0105279

**Published:** 2014-08-29

**Authors:** Ninoa Malki, Ilona Koupil, Sandra Eloranta, Caroline E. Weibull, Sanna Tiikkaja, Erik Ingelsson, Pär Sparén

**Affiliations:** 1 Department of Medical Epidemiology and Biostatistics, Karolinska Institutet, Stockholm, Sweden; 2 Centre for Health Equity Studies (CHESS), Stockholm University/Karolinska Institutet, Stockholm, Sweden; 3 Department of Medical Sciences, Molecular Epidemiology and Science for Life Laboratory, Uppsala University, Uppsala, Sweden; Universität Bochum, Germany

## Abstract

**Background:**

We analyzed temporal trends in the incidence of myocardial infarction and ischemic stroke in Sweden by socioeconomic position and investigated whether social inequalities in incidence of these diseases changed over time.

**Materials and Methods:**

We studied a cohort of almost three million Swedish residents born between 1932 and 1960 followed from 1987 until 2010. Incident cases of myocardial infarction and ischemic stroke were identified in the Swedish National Inpatient Register and Cause of Death Register. Socioeconomic position was retrieved from the Population and Housing Censuses. Incidence rates of myocardial infarction and ischemic stroke and incidence rate ratios comparing levels of socioeconomic position were estimated using flexible parametric survival models adjusted for calendar year, attained age, sex, and birth country.

**Results:**

The overall incidences of myocardial infarction and ischemic stroke decreased over time among men, but were stable over time among women. With regard to ischemic stroke incidence, socioeconomic inequality increased over time in the age group 55 to 59: the incidence rate ratios for low manual compared to high non-manual increased from 1.3 (95% CI: 1.2–1.4) in 1997 to 1.5 (1.4–1.7) in 2010 among men, and from 1.4 (1.3–1.6) in 1997 to 2.1 (1.8–2.5) in 2010 among women. The socioeconomic inequality in incidence of myocardial infarction was stable over time for both men and women.

**Conclusion:**

There was a decrease in myocardial infarction and ischemic stroke incidence over time among men but no significant change for women. Our study highlights existing, and in some cases increasing, social inequalities in the incidence of cardiovascular diseases.

## Introduction

In high income countries, incidence of cardiovascular diseases (CVD) has declined since the 1970s. Nevertheless, CVD is the most common cause of death among both men and women, accounting for over 40 percent of total mortality [1]. In Sweden there occurs annually almost 40,000 incident cases of myocardial infarction (MI) and 30,000 incident cases of stroke, of which more than 20,000 are ischemic strokes (IS) [2]. Previous studies from Sweden have reported a decreasing trend in incidence of MI and stroke among men but inconsistent results among women [3], [4], [5]. Generally, an inverse relationship has been reported between social class and CVD incidence for both men and women [6], [7], [8], [9], with a doubled risk among the manual labor class compared to the non-manual labor class [6].

Several studies have reported a widening over time of relative socioeconomic inequalities in CVD mortality for many European countries, including the Nordic countries [10], [11]. However, results are inconsistent for CVD incidence [12], [13], [14]. Kunst et al. emphasizes the importance of measuring both relative and absolute differences when monitoring trends in socioeconomic inequalities [15].

The primary aim of this study was to analyze temporal trends of MI and IS in both absolute and relative terms across different strata of socioeconomic position (SEP) in Sweden. A secondary aim was to compare the observed trends among men and women, and in different age groups. Using population-based register information, we were able to analyze the temporal variations in different age-groups and using a more finely graded representation of SEP (5 levels) than usual (2 levels).

## Materials and Methods

### Ethics Statement

Ethical approval was obtained from the Regional Ethical Review Board, Karolinska Institutet, Sweden.

### Population

The study cohort consisted of all Swedish residents (Figure S1) born between 1932 and 1960 who had information on SEP in the mandatory Population and Housing Census 1990. For individuals not registered in the 1990 Census, information about SEP was retrieved from 1980 Census (<10% of the cohort). The Census questionnaires were sent to all nationally registered Swedish residents, who were required by law to respond. The response rate was 99% in 1980 [16] and 98% in 1990 [17].

The start of follow-up was set to January 1, 1987 or age 30 for those born after January 1957. The study cohort was linked to the Swedish National Inpatient Register (IPR), and those with a diagnosis of MI or IS prior to start of follow-up were excluded (n = 12,740). Study participants were followed until a diagnosis of MI/IS or censoring (date of death, first emigration or end of follow-up at December 31, 2010), whichever came first. Individuals who immigrated after January 1, 1987 at age 30 years or older were excluded because it was not possible to ascertain if they were disease-free at the start of follow-up (<1%). We lacked register information on emigrations after December 2002. In total, 2,939,771 individuals were included in the study cohort.

### Data sources

The Total Population Register in Sweden (TPR) was established in 1961 and contains all individuals born since1932, and the parents of those individuals who were resident and registered in Sweden in 1961 or later [18]. The IPR registers all hospital discharges with overnight stay in Swedish counties and has partial national coverage from 1964 and full national coverage from 1987 [19]. The Cause of Death Register (CDR) had full national coverage from 1961, including all deaths (99.5%) among nationally registered Swedish residents [20]. The IPR and CDR were used to identify the incidence of MI and IS as the first primary diagnosis upon discharge (non-fatal) or primary cause of death (fatal) between January 1, 1987 and December 31, 2010. The diagnostic validity and coverage of MI and IS in the IPR and CDR is considered good [19], [21], [22], [23]. MI was identified according to the International Classification of Diseases (ICD) with ICD-9 codes 410 (years 1987–1996) and ICD-10 codes I21, I22 (years 1997–2010). IS was identified with ICD-9 codes 433–434 and ICD-10 code I63. ICD-8 codes 410, 432–434 (years 1969–1986) were used to exclude all individuals who had MI or IS before January 1, 1987. The Immigration (from 1969) and Emigration Registers (from 1961) were used to identify first emigration date from Sweden until 2002, and first immigration date for non-Swedish born individuals.

SEP was derived from the Socioeconomic Index (SEI) [24], as coded in the 1990. The Swedish SEI classification was constructed in the 1970s [25] by professor Robert Erikson and colleagues which by and large corresponds to Erikson, Goldthorpe and Portocarrero classification of occupations (EGP) [26]. The SEI classification distinguishes between employers and employees. According to SEI, employees were divided into manual and non-manual occupations (based on union affiliation). The manual and non-manual occupations were then further divided into high and low, according to the required educational level for the occupation in question [24]. SEI classification also takes into consideration job responsibility level, and specific duties or work tasks to be performed. Occupational class (SEI) is considered a good social indicator for individuals above 30 years [27].

We classified SEP in five different groups: high non-manual (HN-M; SEI = 46, 56, 57), low non-manual (LN-M; SEI = 33, 36), self-employed including farmers (SE; SEI = 60, 79, 89), high manual (HM; SEI = 21, 22) and low manual (LM; SEI = 11, 12). Information on sex, birth date, and birth country (Sweden, other Nordic country, other European country, other country) was retrieved from TPR.

### Statistical methods

We used flexible parametric survival models [28] to estimate incidence rates (IR) per 100,000 person-years and incidence rate ratios (IRR) of MI and IS by SEP. These models are conceptually similar to Cox models but use restricted cubic splines to explicitly model the chosen time scale. Calendar year was used as the primary time scale (modeled continuously using 5 degrees of freedom for the spline function), and attained age as a secondary time-scale (via stratification on attained age groups; 45–54 years, 55–59 years, 60–64 years and 65–69 years). Due to low number of incident cases in ages <45, younger individuals were not included in the regression models but only in descriptive analysis. The exposure of interest was SEP. Crude, adjusted, and age- and sex standardized IR were calculated. The latter were obtained by direct standardization using the age and sex distribution in the Swedish population in 2011 as weights.

All regression models were further stratified by sex and attained age, and adjusted for birth country. In addition, interaction effects between the variables were formally tested using likelihood ratio tests. The proportional hazards assumption for the effect of SEP on MI and IS was relaxed by including interaction terms between the variable representing SEP and the restricted cubic spline terms representing the main time scale (i.e. calendar year). These interaction effects were not all statistically significant (as formally tested using LR-tests), but were still kept in the models as we did not want to force a constant relative relationship between the levels of SEP. Since our follow-up started in 1987 (when the IPR became nationwide), all age groups under study were not fully represented over all calendar years in the first years of follow-up (Figure S2). In the age-stratified graphs presenting incidence of MI and IS for SEP groups over calendar time, the years without full age-group representation were indicated by shading.

Data preparation was carried out using SAS 9.3 software (SAS Institute Inc., Cary, NC, USA). STATA (Stata Statistical Software: Release 12. College Station, TX: StataCorp LP) was used for the statistical analyses.

## Results

A total of 121,496 MI cases (83% non-fatal, 17% fatal) and 61,421 IS cases (96% non-fatal, 4% fatal) were registered from 1987 to 2010. The age- and sex-standardized IR of MI and IS are presented for different SEP in Table 1, together with crude IR of MI and IS by sex, birth country and attained age. The IR for both MI and IS were highest in the LM group and lowest in the HN-M group (Table 1). The risk of developing MI and IS was consistently higher among men as compared to women (Table 1). Swedish-born individuals had a lower risk of MI and IS than immigrants from European countries (Table 1). A sensitivity analysis addressing the lack of emigration information from 2002 onwards showed that about 1% of the cohort would emigrate during the eight years missing information and that emigration was equally distributed among SEP groups. For age group 45–54 years old, the results for both MI and IS were very similar to the older age groups (data not shown).

**Table 1:** Frequencies and incidence rates of myocardial infarction and ischemic stroke by sex, attained age, birth country and socioeconomic position.

		Myocardial infarction		Ischemic stroke	
	N	Cases	Incidence rate^1^ (95% CI)	Cases	Incidence rate^1^ (95% CI)
**Total**	2,939,771	121,496	193 (192–194)	61421	97 (96–98)
**Sex**					
Male	1,516,604	94,264	294 (292–296)	40611	125 (124–127)
Women	1,423,167	27,232	88 (87–89)	20810	67 (66–68)
**Attained age**					
30–44 years	2,115,760	5,959	33 (32–34)	2245	12 (12–13)
45–54 years	2,884,922	33,933	146 (144–147)	12684	54 (53–55)
55–59 years	2,303,533	28,798	284 (281–288)	13335	130 (128–133)
60–64 years	1,743,008	29,191	404 (400–409)	17037	233 (229–236)
65–69 years	1,132,923	23,615	559 (552–566)	16120	375 (369–380)
**Birth country**					
Sweden	2,635,227	107,443	189 (188–190)	54367	95 (94–96)
Other Nordic country	148,735	7,232	242 (237–248)	3964	132 (128–136)
Other European country	98,951	4,634	228 (222–235)	2280	112 (107–116)
Other	56,74	2,182	185 (177–193)	809	68 (63–73)
Missing	118				
		**Cases**	**Incidence rate^2^ (95% CI)**	**Cases**	**Incidence rate^2^ (95% CI)**
**Socioeconomic status**					
High Non-manual	933,901	31,763	305(302–309)	16,524	173(170–176)
Low Non-Manual	477,911	15,754	402(395–409)	9,026	215(210–220)
Self-Employee	223,359	12,270	413(405–421)	5,978	226(220–232)
High Manual	479,365	24,042	433(426–439)	10,997	229(224–234)
Low Manual	825,235	37,667	497(492–503)	18,896	252(249–256)

1 Unadjusted incidence rate per 100,000 person-years.

2 Age and sex standardized incidence rate per 100,000 person-years using the Swedish population in 2011 as standard population).

### Myocardial infarction

**Figure 1:**
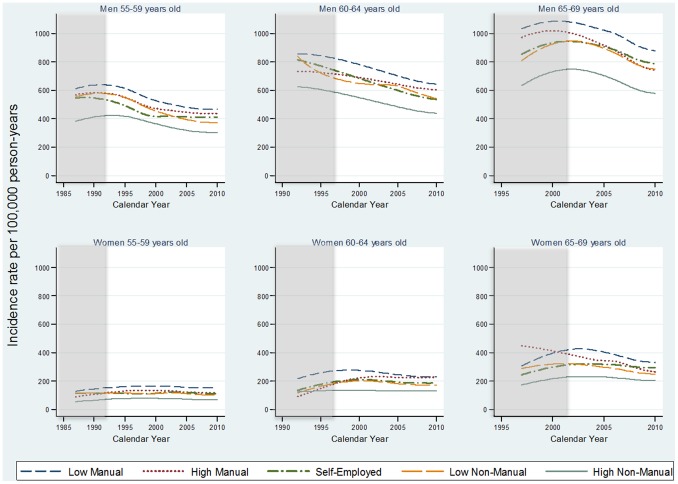
Incidence rates of myocardial infarction by socioeconomic position for Swedish men and women in three age groups. All models were adjusted for birth country and stratified by sex and attained age. Note 1 Figure 1: The shadowed area indicates a time period for which results cannot be interpreted.

There were large socioeconomic inequalities in the incidence of MI for both men and women, across all age groups (Figure 1). The HN-M group had the lowest incidence followed by LN-M, SE, HM and LM. The estimated IR of MI from the attained age- and sex-stratified model, decreased in a similar manner over calendar years among men across all SEP- and age groups (Figure 1). For example among men in the LM group, aged 55 to 59 years, the IRs per 100,000 person-years decreased from 639 (95% CI: 604–675) in 1991 to 464 (95% CI: 441–489) in 2010. The corresponding decrease among men in the HN-M group went from 417 per 100,000 person-years (95% CI: 394–442) in 1991 to 302 per 100,000 person-years (95% CI: 287–318) in 2010 (Table S1). Among Swedish women the incidence was stable over time for all SEP- and age groups. Among women aged 55 to 59 years, the IRs in 1991 were 148 per 100,000 person-years (95% CI: 135–162) and 152 per 100,000 person-years (95% CI: 140–165) in 2010 for the LN-M group (Table S1). Women in the HN-M group had an IR of 67 per 100,000 person-years in both 1991 and 2010 (95% CI: 56–80 and 60–76 respectively) (Table S1). After 2008, the decreasing trend among men seemed to stabilize and among women there seemed to be a moderate increase for all SEP- and age groups.

The IRRs, comparing every SEP group with the HN-M group were stable over time for both men and women (Table 2). The IRR in MI incidence was larger for women than for men; among women the IRR in MI in 2010 was 2.2 (95% CI: 1.8–2.7) when comparing LM with HN-M groups and correspondingly among men it was 1.5 (95% CI: 1.4–1.7).

**Table 2:** Incidence rate ratios (IRR) with 95% confidence intervals (CI) of myocardial infarction and ischemic stroke by socioeconomic position and calendar year, and stratified by sex and attained age.

			Myocardial infarction					Ischemic stroke				
			IRR (95% CI)					IRR (95% CI)				
	Age	Calendar year	High Non-manual	Low Non-Manual	Self- Employed	High Manual	Low Manual	High Non-manual	Low Non-Manual	Self-Employed	High Manual	Low Manual
Men	55–59 years	1991	1.00	1.4 (1.3–1.5)	1.3 (1.2–1.4)	1.4 (1.3–1.5)	1.5 (1.4–1.7)	1.00	1.2 (1.0–1.5)	1.1 (0.9–1.3)	1.3 (1.1–1.5)	1.3 (1.1–1.5)
		1997	1.00	1.3 (1.2–1.4)	1.1 (1.0–1.2)	1.3 (1.2–1.4)	1.4 (1.4–1.5)	1.00	1.3 (1.1–1.4)	1.1 (1.0–1.2)	1.2 (1.1–1.3)	1.3 (1.2–1.4)
		2010	1.00	1.2 (1.1–1.3)	1.4 (1.2–1.5)	1.4 (1.3–1.5)	1.5 (1.4–1.6)	1.00	1.4 (1.2–1.6)	1.5 (1.2–1.7)	1.4 (1.3–1.6)	1.5 (1.4–1.7)
	60–64 years	1997	1.00	1.2 (1.1–1.3)	1.3 (1.2–1.4)	1.2 (1.1–1.3)	1.4 (1.3–1.5)	1.00	1.2 (1.0–1.3)	1.3 (1.2–1.4)	1.3 (1.2–1.4)	1.4 (1.3–1.6)
		2005	1.00	1.3 (1.2–1.4)	1.2 (1.2–1.3)	1.3 (1.3–1.4)	1.5 (1.4–1.5)	1.00	1.3 (1.2–1.4)	1.3 (1.2–1.4)	1.3 (1.2–1.4)	1.4 (1.3–1.5)
		2010	1.00	1.2 (1.1–1.4)	1.2 (1.1–1.3)	1.4 (1.3–1.5)	1.5 (1.4–1.6)	1.00	1.3 (1.1–1.5)	1.2 (1.1–1.4)	1.3 (1.2–1.5)	1.4 (1.3–1.6)
	65–69 years	2002	1.00	1.3 (1.2–1.4)	1.3 (1.2–1.4)	1.3 (1.2–1.4)	1.4 (1.3–1.5)	1.00	1.2 (1.1–1.3)	1.3 (1.2–1.5)	1.3 (1.2–1.4)	1.3 (1.2–1.4)
		2005	1.00	1.3 (1.2–1.4)	1.3 (1.2–1.4)	1.3 (1.2–1.4)	1.5 (1.4–1.5)	1.00	1.1 (1.0–1.3)	1.3 (1.2–1.4)	1.3 (1.2–1.4)	1.4 (1.3–1.5)
		2010	1.00	1.3 (1.2–1.4)	1.4 (1.3–1.5)	1.3 (1.2–1.4)	1.5 (1.4–1.6)	1.00	1.2 (1.0–1.3)	1.3 (1.1–1.4)	1.2 (1.1–1.3)	1.5 (1.3–1.6)
Women	55–59 years	1991	1.00	1.7 (1.4–2.1)	1.7 (1.3–2.3)	1.7 (1.2–2.2)	2.2 (1.8–2.7)	1.00	1.2 (0.9–1.5)	1 (0.6–1.5)	1.4 (1–2)	1.2 (0.9–1.5)
		1997	1.00	1.4 (1.2–1.6)	1.4 (1.1–1.8)	1.7 (1.4–2.0)	2.1 (1.8–2.3)	1.00	1.2 (1.1–1.4)	1.2 (1–1.5)	1.5 (1.3–1.7)	1.4 (1.3–1.6)
		2010	1.00	1.5 (1.3–1.8)	1.6 (1.2–2.2)	1.7 (1.4–2.1)	2.2 (2.0–2.6)	1.00	1.6 (1.3–1.9)	1.4 (1–2)	1.8 (1.4–2.2)	2.1 (1.8–2.5)
	60–64 years	1997	1.00	1.4 (1.2–1.6)	1.5 (1.2–1.8)	1.4 (1.1–1.7)	2.0 (1.8–2.3)	1.00	1.4 (1.2–1.6)	1.4 (1.1–1.8)	1.3 (1.1–1.7)	1.4 (1.2–1.6)
		2005	1.00	1.4 (1.3–1.5)	1.5 (1.3–1.7)	1.7 (1.6–1.9)	1.9 (1.7–2.0)	1.00	1.2 (1.1–1.4)	1.3 (1.1–1.5)	1.4 (1.2–1.6)	1.5 (1.4–1.6)
		2010	1.00	1.3 (1.2–1.5)	1.4 (1.2–1.7)	1.7 (1.5–2.0)	1.7 (1.6–1.9)	1.00	1.2 (1.1–1.4)	1.3 (1–1.6)	1.3 (1.1–1.6)	1.5 (1.3–1.7)
	65–69 years	2002	1.00	1.4 (1.2–1.6)	1.4 (1.1–1.6)	1.7 (1.4–2.0)	1.8 (1.7–2.0)	1.00	1.2 (1–1.3)	1.5 (1.2–1.7)	1.2 (1–1.5)	1.4 (1.3–1.6)
		2005	1.00	1.3 (1.2–1.5)	1.4 (1.2–1.6)	1.5 (1.3–1.8)	1.8 (1.6–2.0)	1.00	1.2 (1–1.4)	1.5 (1.2–1.7)	1.2 (1–1.4)	1.4 (1.3–1.6)
		2010	1.00	1.2 (1.1–1.4)	1.4 (1.2–1.8)	1.3 (1.1–1.6)	1.6 (1.4–1.8)	1.00	1.3 (1.1–1.5)	1.3 (1.1–1.6)	1.5 (1.1–1.9)	1.5 (1.4–1.8)

Corresponding analyses were performed for ischemic heart disease, (ICD-9 codes 410, 411.1 and ICD-10 codes I20.0, I21, I22), yielding results very similar to MI (Figure S3).

### Ischemic stroke

**Figure 2:**
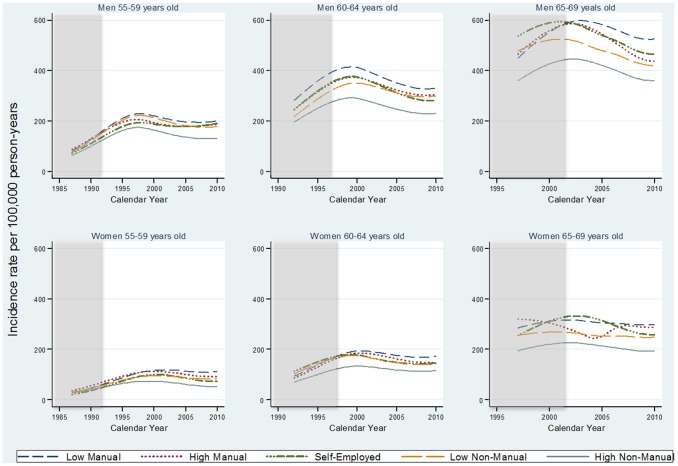
Incidence rates of ischemic stroke by socioeconomic position for Swedish men and women in three age groups. All models were adjusted for birth country and stratified by sex and attained age. Note 1 Figure 2: The shadowed area indicates a time period for which results cannot be interpreted. Note 2 Figure 2: The incidence rate of ischemic stroke is increasing until 1997 due to changing in ICD codes 9 and 10, the result until 1997 is uncertain.

Large socioeconomic inequalities were seen for men and women and across all age groups in the incidence of IS (Figure 2). The largest socioeconomic differences were observed between HN-M and LM groups. The other socioeconomic groups (LN-M, SE, HM) had a quite similar incidence of IS, in-between that of HN-M and LM. For men, the incidence of IS decreased from 1997 and onwards in all age groups for the HN-M group while the LM showed a moderate, but not significant, decrease. Among men in the LM group, aged 55 to 59, the IR per 100,000 person-years was 227 (95% CI: 213–243) in 1997 and 200 (95% CI: 183–218) in 2010, while the corresponding IR among men in the HN-M group was 173 (95% CI: 163–184) and 131 (95% CI: 120–143) (Table S1). Women in the same age groups, belonging to the LM group displayed IR per 100,000 person-years of 102 (95% CI: 95–111) in 1997 and 111 (95% CI: 100–124) in 2010 (Table S1). Women in the HN-M group displayed corresponding IR of 71 (95% CI: 64–79) and 52 (95% CI: 45–61) (Table S1). For the last 3 to 5 years of the follow-up the trend in incidence of IS seemed to increase for all SEP- and age groups, both among men and women (Figure 2).

The IRR for IS, comparing LM to HN-M, changed over time. Among men aged 55 to 59 (Table 2) the IRR was 1.3 (95% CI: 1.2–1.4) in 1997 and 1.5 (95% CI: 1.4–1.7) in 2010. Even more pronounced, among women the IRR increased from 1.4 (95% CI: 1.3–1.6) in 1997 to 2.1 (95% CI: 1.8–2.5) in 2010. In the older age group (60 to 64; 65 to 69) IRR, comparing LM with HN-M, seemed to increase both among men and women, while the trend was not as obvious as in the younger age group.

## Discussion

In the present study, we showed that the incidence of MI declined over time among men but not among women. However, this declining incidence of MI among men seemed to diminish in the last years of follow-up. IS incidence also did not decline among women in any SEP group, and among men in the LM group. The socioeconomic inequalities in incidence of MI and IS did not decrease over time in both sexes. In fact, among younger men and women there were increasing social inequalities over time in IS.

Few studies have analyzed the temporal changes of social inequality in MI and IS incidence. Our findings of no changes in social inequalities in MI incidence parallel those by a Finnish study [13]. Other studies have shown increasing social inequalities in ischemic heart disease morbidity for certain occupations [12], [14].

Among both men and women ages 55 to 59 in the HN-M group, the trend in incidence in IS decreased since 1997. For the LM group the decreasing trend was not as obvious for men and particularly not for women, which resulted in increasing socioeconomic inequalities. This may indicate that the HN-M group adopts healthier lifestyles such as smoking cessation earlier than the LM group [29].

Several factors may influence the observed persisting socioeconomic inequalities in our study. It is well known that risk factors as cigarette smoking, hypertension, cholesterol, obesity, type-2 diabetes and lack of physical activity explain a large part of the association between socioeconomic inequalities and cardiovascular morbidity [30]. Over time, socioeconomic inequality with regards to smoking and obesity has increased, while it has remained stable with regards to cholesterol and type-2 diabetes [31], [32]. We found that the IRR in MI incidence was larger among women than for men when comparing LM to HN-M, which was in line with previous study from Sweden [5]. This difference could be due to larger socioeconomic inequalities of several CVD risk factors among women. Relative inequalities in smoking and hypertension were found to be significantly higher among women than among men [31].

Previous Swedish studies have reported a decreasing trend in incidence for MI in men, but inconsistent results for women [3], [5], [33]. The MI incidence decline among men but not among women seen in our data may also be explained by common CVD risk factors. Generally, the decline of smoking is less obvious among women than among men in most Western countries [34]. A recent study in Sweden has reported a modest decline in smoking rates and a marked increase in obesity, triglyceride levels and experiences of stress among middle-aged women [35]. Also high blood pressure has increased among younger women [31].

Previous studies from Sweden observed a lower risk of MI and IS among Swedish born compared to immigrants from European countries [5], [36] This is in line with our results, but since those studies did not focus on the effect of socioeconomic position, we believe that our analysis make an important contribution to this area.

The reason that IS incidence increased until 1997 and declined afterwards is unclear. Earlier reports from Sweden presented an increasing IS incidence during the 1990s [4], [37], [38]. The observation may be partially explained by better diagnosis over time. Medin et al [37] reported that undetermined stroke decreased over time in Sweden while IS has increased. Also, the ICD-code classification changed in 1997. The ICD-10 classification of IS is more specific than the previous one. It is possible that the true underlying trend in IS is decreasing, which is then picked up with the ICD-10 classification in 1997. Therefore we decided to interpret results for IS beginning in 1997.

### Strengths and Limitations

Strengths of this study include the population-based design, the large number of events, and our improved measure of SEP.

Several different indicators of SEP are used in epidemiology [25]. The most common measures are based on occupation, education, income, or a combination of these [25]. The classification used in the present study is the socioeconomic index (SEI) based on occupation, but it also takes education needed for a specific occupation into account. The occupation was reported by subjects themselves in 1980 or 1990. Record linkage of the data at individual level provided us with accurate information on SEP prior to the event (only 1.5% and 3% of all MI and IS cases in our study, respectively, measured the SEP after diagnosis date). One advantage of this study is that the SEP could be grouped, not only into manual and non-manual, but also subdivided into more finely graded five SEP groups. This allowed us to separate LM from HM, who showed to by a valuable group. Individuals missing occupational information were excluded from the study, since they comprise a heterogeneous group including housewives, students, unemployed, disabled individuals and those early retired. We do however know that unclassifiable individuals have the highest risk in cardiovascular mortality compared to other SEP groups [39].

One limitation is that we were not able to represent all age groups in every year. In our cohort, individuals were born between 1932 and 1960 and followed from 1987 to 2010. Over the calendar years, all age groups were not fully representative over the total age distribution in the Swedish population (Figure S2). For example, in 1990 the oldest person in our cohort was 58 years old; therefore, we cannot interpret the results before 1991 for the age group 55 to 59, because the age group is not fully representative. We presented these calendar years for different age groups as shaded area in the figures and do not include those results in our interpretation.

In Sweden, the mean age for IS incidence is 73 years for men and 79 years for women. Another limitation is that our study does not reflect the total burden of MI and IS in the Swedish population, since our study population only includes individuals born 1932 or later, and we therefore could not include any person older than 78 years old in our study.

Changes in diagnostic practice over time are potential sources of bias in long-term incidence studies. With the increased use of cardiac enzymes to diagnose MI and the introduction of computed tomographic scans to diagnose stroke, the quality and quantity of information available to the practicing physician has changed over time. Although access to health care is theoretically equal across SEP groups in the publically funded Swedish system, there might still be differences in diagnosis between SEP groups, for example due to differences in attitudes towards seeking health care, reactions to symptoms, abilities to express and explain symptoms and need for immediate care, or geographical obstacles.

Although we tried to avoid reverse causation by excluding individuals with MI or IS prior 1987, there is still a risk that we included a few individuals with prior disease as the Swedish IPR had full coverage only after1987. However, the coverage was as high as 88% from 1983 [19] and the studied population was young (MI and IS are rare diseases in the younger population). Hence, reverse causation arising from individuals experiencing an event which might cause lower SEP should not have affected our findings to any large extent.

## Conclusions

The incidence of MI in men decreased over time while it was stable for women. The large socioeconomic inequality in MI and IS incidence persisted over the study period, and in younger individuals there was even a tendency of increasing inequality in IS incidence. Further research needs to establish the impact of life style, psychosocial factors and preventative health services on existing inequalities in order to design more effective and gender specific health policies.

## Supporting Information

Figure S1 **Study cohort graph.**
(TIF)

Figure S2 **Study cohort age distribution.**
(TIF)

Figure S3 **Incidence rates of ischemic heart diseases by socioeconomic position for Swedish men and women in three age groups.** All models were adjusted for birth country and stratified by sex and attained age. Note 1 Figure S3: The shadowed area indicates a time period for which results cannot be interpreted.
(TIF)

Table S1 **Incidence rate (IR) with 95% confidence intervals (CI) of myocardial infarction and ischemic stroke by socioeconomic position and calendar year, and stratified by sex and attained age.**
(DOCX)
