# Supplementary figures and images for: Temporal Trends in Incidence of Myocardial Infarction and Ischemic Stroke by Socioeconomic Position in Sweden 1987–2010
Source: PLoS One. 2014 Aug 29;9(8):e105279. doi: 10.1371/journal.pone.0105279 (PMC4149372; doi:10.1371/journal.pone.0105279)

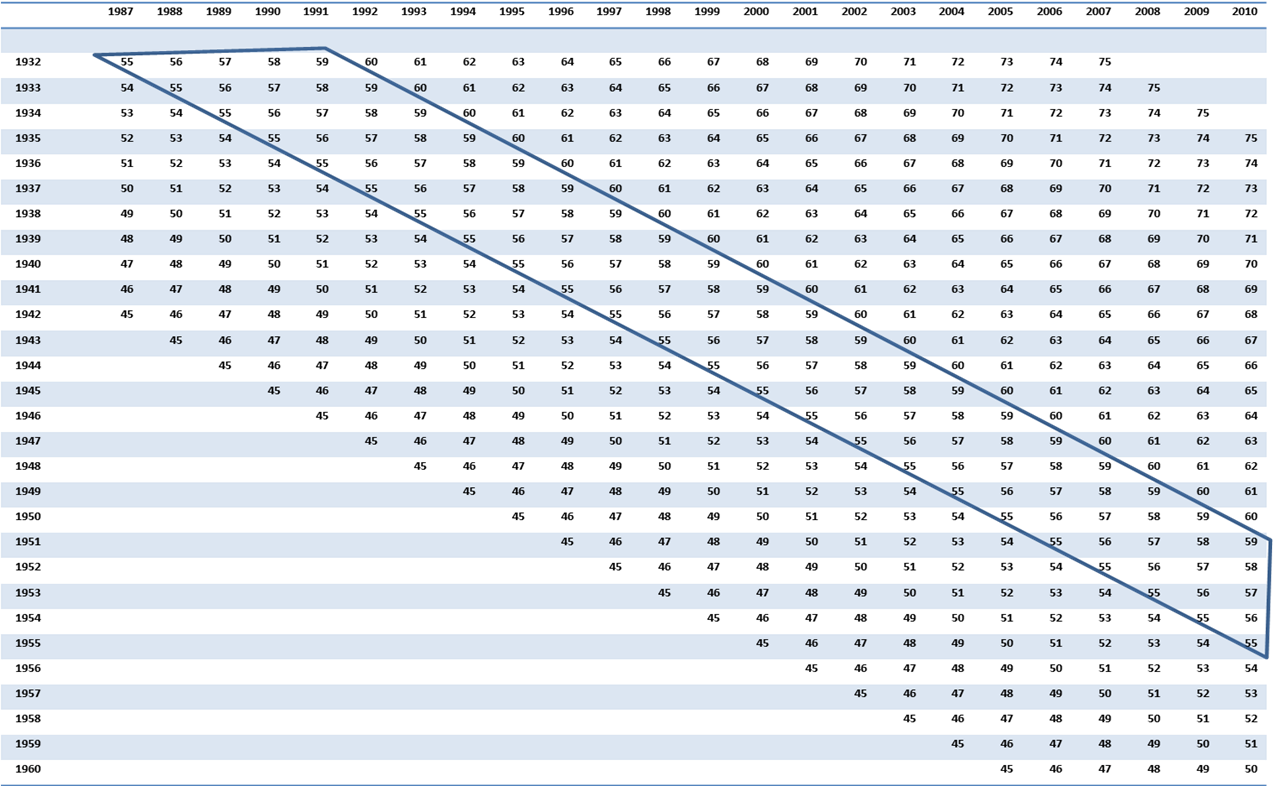

Supplement: Figure S2 — Study cohort age distribution. (TIF) [file pone.0105279.s002.tif]

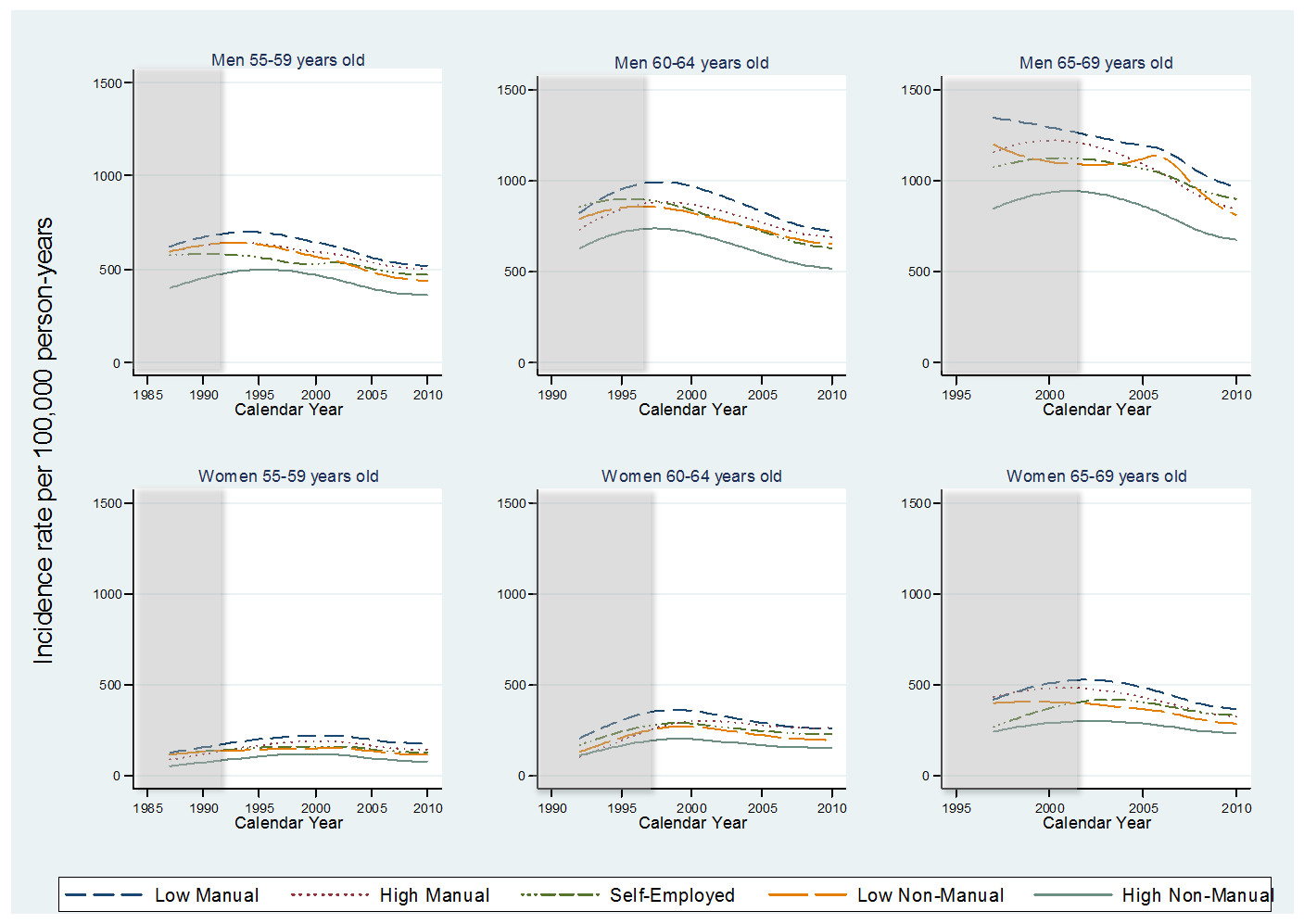

Supplement: Figure S3 — Incidence rates of ischemic heart diseases by socioeconomic position for Swedish men and women in three age groups. All models were adjusted for birth country and stratified by sex and attained age. Note 1 Figure S3: The shadowed area indicates a time period for which results cannot be interpreted. (TIF) [file pone.0105279.s003.tif]
