# Supplementary material for: Temporal Trends in Incidence of Myocardial Infarction and Ischemic Stroke by Socioeconomic Position in Sweden 1987–2010
Source: PLoS One. 2014 Aug 29;9(8):e105279. doi: 10.1371/journal.pone.0105279 (PMC4149372; doi:10.1371/journal.pone.0105279)
Supplement: Table S1 — Incidence rate (IR) with 95% confidence intervals (CI) of myocardial infarction and ischemic stroke by socioeconomic position and calendar year, and stratified by sex and attained age. (DOCX) [file pone.0105279.s004.docx]

**Table 2**: Incidence rate (IR) with 95% confidence intervals (CI) of myocardial infarction and ischemic stroke by socioeconomic position and calendar year, and stratified by sex and attained age.

|  |  |  | Myocardial infarction | | | | | Ischemic stroke | | | | |
| --- | --- | --- | --- | --- | --- | --- | --- | --- | --- | --- | --- | --- |
|  |  |  | IR (95% CI) | | | | | IR (95% CI) | | | | |
|  | Age | Calendar year | High Non-manual | Low Non-Manual | Self- Employed | High Manual | Low Manual | High Non-manual | Low Non-Manual | Self-Employed | High Manual | Low Manual |
| Men | 55-59 years | 1991 | 417 [394-442] | 578 [532-627] | 539 [497-585] | 582 [547-619] | 639 [604-675] | 113 [101-126] | 138 [117-163] | 125 [106-147] | 144 [127-162] | 145 [130-162] |
|  |  | 1997 | 398 [383-413] | 510 [476-546] | 451 [419-484] | 513 [488-540] | 577 [551-603] | 173 [163-184] | 220 [201-242] | 192 [175-211] | 205 [191-221] | 227 [213-243] |
|  |  | 2010 | 302 [287-318] | 370 [340-402] | 410 [378-444] | 435 [412-459] | 464 [441-489] | 131 [120-143] | 178 [156-204] | 190 [167-218] | 187 [171-205] | 200 [183-218] |
|  | 60-64 years | 1997 | 583 [559-609] | 680 [634-730] | 736 692-783] | 713 [678-750] | 822 [786-859] | 277 [260-296] | 325 [294-359] | 357 [326-390] | 353 [328-379] | 396 [372-423] |
|  |  | 2005 | 483 [469-498] | 628 [572-690] | 598 [569-628] | 641 [618-665] | 701 [676-727] | 246 [234-259] | 312 [289-336] | 312 [290-335] | 322 [305-341] | 351 [333-370] |
|  |  | 2010 | 436 [418-455] | 541 [489-598] | 534 [499-572] | 602 [573-632] | 642 [612-673] | 230 [215-246] | 299 [267-335] | 281 [252-314] | 305 [282-329] | 329 [305-355] |
|  | 65-69 years | 2002 | 747 [712-784] | 945 [873-1023] | 944 [883-1008] | 999 [941-1060] | 1077 [1019-1138] | 444 [419-471] | 520 [474-570] | 589 [542-640] | 585 [547-624] | 592 [557-628] |
|  |  | 2005 | 702 [675-730] | 894 [833-959] | 905 [853-960] | 916 [872-962] | 1022 [977-1068] | 420 [398-443] | 478 [434-527] | 539 [492-590] | 543 [506-583] | 581 [547-618] |
|  |  | 2010 | 577 [548-606] | 748 [686-814] | 787 [730-848] | 739 [695-786] | 876 [827-927] | 359 [337-384] | 419 [374-470] | 463 [419-513] | 436 [401-473] | 525 [488-565] |
| Women | 55-59 years | 1991 | 67 [56-80] | 115 [101-130] | 114 [91-143] | 111 [88-140] | 148 [135-162] | 45 [36-56] | 52 [43-63] | 43 [29-63] | 63 [46-85] | 53 [46-62] |
|  |  | 1997 | 79 [71-87] | 111 [101-122] | 111 [91-135] | 133 [117-152] | 162 [151-174] | 71 [64-79] | 88 [80-97] | 88 [73-106] | 105 [91-121] | 102 [95-111] |
|  |  | 2010 | 67 [60-76] | 101 [89-115] | 110 [84-143] | 117 [100-136] | 152 [140-165] | 52 [45-61] | 83 [72-95] | 72 [52-101] | 92 [76-111] | 111 [100-124] |
|  | 60-64 years | 1997 | 134 [119-150] | 184 [168-203] | 195 [166-230] | 183 [157-215] | 272 [255-291] | 119 [105-134] | 163 [147-180] | 169 [141-203] | 158 [133-189] | 167 [155-180] |
|  |  | 2005 | 131 [121-140] | 181 [170-193] | 192 [171-216] | 225 [207-246] | 243 [231-257] | 118 [108-128] | 147 [135-159] | 148 [128-171] | 161 [143-181] | 175 [164-187] |
|  |  | 2010 | 131 [119-143] | 170 [155-186] | 184 [154-220] | 228 [200-259] | 228 [212-246] | 115 [103-128] | 141 [127-157] | 145 [116-181] | 149 [126-176] | 172 [157-188] |
|  | 65-69 years | 2002 | 230 [209-253] | 319 [292-348] | 317 [270-370] | 381 [330-439] | 423 [400-447] | 225 [203-250] | 266 [242-292] | 331 [288-381] | 278 [236-329] | 315 [293-339] |
|  |  | 2005 | 228 [210-247] | 297 [276-320] | 317 [275-366] | 343 [298-395] | 404 [383-426] | 213 [197-230] | 252 [219-290] | 314 [270-364] | 250 [210-298] | 304 [280-331] |
|  |  | 2010 | 204 [185-225] | 248 [225-272] | 294 [247-350] | 263 [222-312] | 329 [307-354] | 192 [175-212] | 248 [222-276] | 257 [212-311] | 286 [224-364] | 298 [275-323] |
